# Insecure messaging: how clinicians approach potentially problematic messages from patients

**DOI:** 10.1093/jamiaopen/ooaa051

**Published:** 2020-12-05

**Authors:** Joy L Lee, Marianne S Matthias, Monica Huffman, Richard M Frankel, Michael Weiner

**Affiliations:** 1 Division of General Internal Medicine and Geriatrics, Department of Medicine, Indiana University School of Medicine, Indianapolis, Indiana, USA; 2 Center for Health Services Research, Regenstrief Institute, Inc., Indianapolis, Indiana, USA; 3 Center for Health Information and Communication, U.S. Department of Veterans Affairs, Veterans Health Administration, Health Services Research and Development Service CIN 13-416, Richard L. Roudebush VA Medical Center, Indianapolis, Indiana, USA

**Keywords:** communication, patient portals, electronic health record, practice guideline

## Abstract

**Objective:**

Secure messaging has become an integrated function of patient portals, but misuse of secure messaging by both patients and clinicians can lead to miscommunication and errors, such as overlooked urgent messages. We sought to uncover variations in clinician approaches and responses to messaging with patients.

**Methods:**

In this two-part study, 20 primary care clinicians (1) composed message responses to five hypothetical patient vignettes and messages and (2) were subsequently interviewed for their perspectives on appropriate circumstances for secure messaging. Messages and interviews were analyzed for themes.

**Results:**

Clinicians have different experiences with, and perceptions of, secure messaging. The messages the clinicians wrote were uniformly respectful, but differed in degrees of patient-centeredness and level of detail. None of the clinicians found their messaging workload to be unmanageable. From the interviews, we found divergent clinician perspectives about when to use secure messaging and how to respond to emotional content.

**Conclusion:**

Clinicians have different opinions about the appropriateness of secure messaging in response to specific medical issues. Our results noted a desire and need for greater guidance about secure messaging. This aspect of informatics education warrants greater attention in clinical practice.

**Practical implications:**

We summarize the types of issues raised by the participants yet to be addressed by existing guidelines. Further guidance from hospitals, professional societies, and other institutions that govern clinician behavior on the appropriateness and effectiveness of delivering care through secure messaging may aid clinicians and patients.

**Lay summary**

Secure messaging, electronic written communication between patients and clinicians via a secure portal, can expand patients’ access to their clinicians outside of in-person visits. Despite its benefits, both clinicians and patients have expressed a desire for more guidance on using secure messaging. In this study, we compare primary care clinicians’ responses to secure messaging vignettes to understand their approaches to messaging. We identified similarities and differences in their understanding of appropriateness, as well as the content of the messages. Some clinicians felt that patients are not always able to determine what is an urgent or appropriate message. Some clinicians’ responses were more detailed and patient-centered than others. These differences may differentially impact patients. Institutions that govern and guide patient and clinician behavior, such as health systems, hospitals, and professional societies, should create guidelines and curricula to address patient and clinician needs.

## INTRODUCTION

Secure messaging—electronic written communication between patients and clinicians via a secure portal—is one way to expand patients’ desired access to healthcare and information from their clinicians. Secure messaging allows patients to communicate with clinicians outside in-person visits. The use of secure messaging has increased dramatically in the last 10 years. Spurred by federal adoption incentives, over 60% of physicians had secure-messaging capabilities by 2015.^1^ In the US Department of Veterans Affairs (VA), an estimated 42% of patients had access to secure messaging through activated personal health record portals by 2017.^2^ Yet even as the availability of secure messaging has increased, detailed guidance on how it should be used is lacking—a concern expressed by both clinicians and patients.^3^

Secure messaging is associated with patient satisfaction.^4^ Patients have reported messaging to be helpful, especially for refilling medications.^5^ Literature on the clinical effects of secure messaging is still emerging^4^^,^^6^^,^^7^ and has found patients’ use associated with improved control of diabetes mellitus.^4^^,^^8^^,^^9^ Receipt of secure messages is also associated with medication adherence.^7^ Yet because secure messaging lacks visual and auditory cues, the tone and meaning of the messages, and the recipients’ understanding, may be more difficult to interpret compared to face-to-face conversations. Furthermore, secure messaging is asynchronous; the time between sending and receiving messages may vary. Because of these features, some clinical situations may be inappropriate for secure messaging.

While limited clinician guidelines for secure messaging exist,^10^ they do not offer suggested best practices regarding the messages’ content, nor do they provide guidance about the appropriateness or inappropriateness of messaging in specific situations. Without such guidelines, clinicians are left to their own judgments. Methods by which clinicians make such judgments, and the consistency of these judgments, are unknown. Given the rise in alternatives to in-person visits, these issues are increasingly important. In this qualitative study, we sought to describe and understand clinicians’ judgments about appropriate circumstances for secure messaging, and variations in how they responded to standardized messages. We present how clinicians respond to messages in areas lacking clear guidance, and highlight variations in responses and experiences.

## METHODS

In this two-part study, clinicians (1) composed responses to hypothetical patient vignettes and messages and (2) were interviewed. The study was approved by the Institutional Review Board.

### Sample

This qualitative study targeted primary care clinicians from a Midwestern VA medical center and a safety-net academic medical center. All attending physicians, nurse practitioners, and registered nurses working at the primary care clinics of the two institutions were eligible for the study. Given that many practices take a team approach to secure messaging, we believed it was important to include different types of clinicians in this study. Participants were informed of the study at clinic meetings, via email, and by referral. Clinicians interested in participating were directed to contact the study team via email. Recruitment continued until theme saturation was reached, a point when gathering fresh data “no longer sparks new theoretical insights, nor reveals new properties of… core theoretical categories.”^11^

### Message exercise and interview

Participants were presented with five clinical vignettes and corresponding secure messages from fictitious patients and were asked whether they would have preferred to respond to the patients by phone or secure message, and asked to compose message responses regardless of preference. The standardized vignettes allowed for comparisons across participants, and removed variations in factors like length and quality of the patient–clinician relationship. Participants were then interviewed about their responses, and their perceptions of appropriateness. The interview also included questions about their messaging experiences and workflow. These semi-structured interviews lasted approximately 30 min.

### Vignettes and message development

The five vignettes and corresponding messages from patients were designed to represent scenarios that lacked clear or specific guidance of how to respond. We adapted vignettes and messages from three sources: published literature,^12^de-identified message exchanges between primary care patients and clinicians, and clinicians’ accounts of messaging. The instruments were iteratively refined for clarity and face validity. The vignettes comprised (1) a patient with chest pain (“Urgent” vignette); (2) a patient requesting anti-depressants (“Depression” vignette); (3) a patient reporting an update about her cough and asking for results of a cholesterol test (“Labs and medication” vignette); (4) a patient whose wife asked for clarification about medications (“Spouse and medication” vignette); and (5) a patient with chronic pain who is upset with his care (“Angry” vignette). See Supplementary Appendix for details.

### Analysis

Secure message responses were analyzed using a hybrid inductive and deductive approach. The hybrid approach used an existing coding scheme developed for secure messages^2^ to assess patient-centeredness based on the messages’ purpose, tone (eg, friendliness on a scale from 1 to 3), and content area (eg, medications or referral), and examined emergent themes using inductive analysis. Data were organized into meaningful units and developed into categories (eg, clinical reasoning) for use in representing a coherent account of participants’ perspectives. Interviews were audio-recorded, transcribed, and analyzed using an immersion and crystallization approach to uncover themes.^13^ The team used NVivo software, V.12 (QSR International Pty Ltd) to organize and code messages and interview transcripts.

## RESULTS

Twenty primary care clinicians participated in the study. Table 1 describes their characteristics. We observed variations in how clinicians approached the secure messages and the content of the messages. Results are presented in order of increasing variation.

**Table 1.** Study participant characteristics

Participant characteristics	Number of participants (*n* = 20)	Percentage
Female	14	70
Role		
Physician (attending)	14	70
Nurse practitioner	3	15
Registered nurse	3	15
Age		
Under 45	12	60
45 and over	8	40
Race		
White	14	70
Asian	5	25
Hispanic, Latino, or Spanish	2	10
Number of messages received a week		
10 or fewer	9	45
11 or more	11	55
Perception of message load		
Not manageable	0	0
Somewhat manageable	11	55
Very manageable	9	45

Respondents reported that the messaging tasks reflected situations and messages that they encounter in their practice, describing messages and vignettes as “pretty true to life” (**C3**, registered nurse, female) and “representative of different kinds of interactions with patients through secure messaging” (**C12**, physician, male). Some participants were the primary person responsible for responding to secure messages, while others delegated the task to other members of their care team. Their workflow differed, but none felt that their messaging workload was unmanageable.

### Vignette 1: Urgency: Patient with chest pain away from home

Primary care clinicians agreed that a telephone response was preferable to secure messaging to respond to patients with urgent medical issues. This vignette elicited the highest level of clinician agreement in both approach (ie, whether to use messaging or telephone to respond) and content. Nearly all of the messages included advice for the patient to go to an emergency department. Some added orienting details for the patient, explaining why the chest pains were a concern, and what to do following the emergency visit. Below is a typical response.

I tried to reach you by phone, but was only able to leave a voicemail. I am VERY concerned about the chest pain that you mention. I STRONGLY suggest that you go to the local ER to get checked out. I know that the VA in Seattle is excellent, and I feel confident that you will get good care there. They can see all your records from here, and I will be able to see the results of any testing they do. Please let me know if and when you go, and I will ask Carol our nurse to try and reach you again later today to check in. (**C4**, physician, female)

In the interviews, some clinicians noted that patients cannot always judge a situation’s urgency, and needed guidance about proceeding. “Sometimes patients don’t know what is concerning and what is not,” one commented, “and […] they say oh, I forgot to mention I have chest pains every time I go up a flight of stairs.” (**C17**, physician, female)

### Vignette 2: New depression symptoms

Primary care clinicians also agreed that secure messaging should not be used to respond to a patient expressing new symptoms of depression. Instead, they would have preferred to call a patient who sent a message about “feeling down.” Regarding content, participants conveyed sympathy and used the word “sorry” in their responses; they also indicated concern about self-harm. Messages varied regarding addressing the patient’s request for anti-depressant medication, and the level of empathy displayed. Two responses below exemplify the differences.

Do you have any thoughts of hurting yourself or others? I would like you to talk to our primary care psychologist. Are you available for a telephone call today? (**C11**, nurse practitioner, female)

I’m sorry to hear this is happening…. Often medication can help symptoms of depression, but I need to talk with you a little further. If you’re having any thoughts of hurting yourself, don’t wait to hear from me. You need to call 911 or seek help immediately. Thanks. (**C12**, physician, male)

### Vignette 3: Labs and medications (maintenance)

Clinicians agreed, to a lesser extent than the previous two circumstances, that messaging could be used to address patient questions about routine laboratory results and medication maintenance. Those who disagreed indicated that the topics were appropriate to discuss over messaging, but that the combination of the two questions made the message too complicated. As one participant explained, “If it’s just one question, I think it can be managed. But [this message had] a lot of things, so I don’t feel that messaging is the best way to communicate it.” (**C4**, physician, female) Degrees of patient-centeredness and detail in the messages varied. One participant, for example, provided a terse response.

Labs are ok. I would recommend that you continue to f/u and take your medications as prescribed. (**C20**, nurse practitioner, female)

In contrast, one participant provided more detailed, though at times technical, information.

Your blood work shows that you are at increased risk of having a cardiovascular event about 21% over the next 10 years. I would recommend a statin which can reduce your risk. The main side effects are muscle weakness/tenderness or liver toxicity. If you develop any of these, you should stop taking the medicine. (**C5**, physician, male)

A third participant wrote in a more relational tone, responding to a patient’s personal details.

That is exciting that you went to visit with your granddaughter! … Your cholesterol was elevated, both your total cholesterol and your LDL (lousy artery clogging kind). It is my recommendation that you start atorvastatin 20 mg daily at bedtime for your cholesterol. This will help reduce the risk of stroke and heart attack. You will need to monitor for all over muscle aches out of the ordinary while you are on this, if this occurs or if you have any other problems please let me know! Let me know where you would like this medication called into. (**C7**, nurse practitioner, female)

The tension between message brevity and personalization came up several times in the interviews. Participants spoke of their decision to acknowledge or overlook personal details a patient may disclose, such as the birth of a grandchild in this example. One physician described his philosophy as limiting the content of messages to only “what is useful.” (**C5**, physician, male) In contrast, a physician saw the mention of a new grandchild as useful for building rapport. “It was important to [the patient],” she said (**C16**, physician, female), “[that’s why] she mentioned it.”

### Vignette 4: Spouse and medications (complex)

Responses ranged widely for a request from a patient’s account by someone other than the patient (eg, a spouse) about medication adjustments related to pain and hypertension. Clinicians lacked consensus on whether to use secure messaging, and on the most appropriate recipient. Some addressed the spouse, others the patient, and some addressed both. Regarding the clinical issue, some addressed the questions directly, while others indicated that the message’s complexity warranted a separate discussion by phone or in person.

This message addressed both patient and spouse, and answered the spouse’s questions while also deferring to having a phone discussion.

Hi Mr. and Mrs. Frank,

I will try and give you a call today to discuss some of the medication changes that we made at the last visit. The diltiazem dose was decreased because it seemed that Mr. Frank was light-headed from this medication, if he is having a problem with this change then we can talk about changing it back. (**C4**, physician, female)

In contrast, some participants were uncomfortable responding directly to a spouse. One stated during the interview, “Unless I know that relationship, that [the spouse is] there often, […] they’re the Power of Attorney, or healthcare representative, I won’t give them any information.” (**C14**, physician female) Likewise, the message below is addressed only to the patient, and did not answer the spouse’s question directly.

Mr. Frank, I recently heard from your wife. I understand that she’s concerned about your health but unfortunately I can’t respond to her concerns because of confidentiality laws. I’d be happy to have you sign a release form next time you are in the office allowing me to speak to her directly about your care. My nurse will contact you to schedule an appointment to discuss the concerns you and your wife have. She is welcome to attend if that is what you wish. (**C13**, physician, female)

In the last example, the clinician addressed the spouse directly, and offered only a telephone call if the spouse felt it necessary.

Ms. Frank,

I am happy to hear from you…. He had mentioned that he was dizzy and his blood pressure was a bit low—so I decreased his blood pressure medication from 240 mg to 180 mg on the Diltiazem. … If you are unclear about his medications, let me know—I can have [my nurse] call you. (**C6**, physician, female)

### Vignette 5: Angry patient

Responding to a message from a patient angry about his pain care produced the greatest variation. Clinicians were split between whether a phone call or message would have been the appropriate response platform. Participants sometimes used the same rationale—ease of response—to justify different approaches. One explained her preference for calling by saying, “They’ve got a lot of stuff that they’re concerned about or mad about, and it just seems easier to call them.” (**C11**, nurse practitioner, female) Conversely, another respondent justified secure messaging, saying, “It’s easier to get it in writing and respond to them in writing than to have to engage with a very difficult person.” (**C2**, registered nurse, female) The content of the responses also revealed variations in several themes, including addressing the patient’s concerns, appeasement, discussion of pain management, and the suggested course of action.

Some clinicians offered immediate actions, such as this nurse.

Hello, I will review your chart then discuss your concerns with [the primary care physician] today, and either call you or secure message you after he has been made aware of your concerns. I am sorry that you are disappointed with your care. (**C2**, registered nurse, female)

Others left it to the patient to follow-up.

I am sorry that our appointment did not meet your needs. We probably should meet again to discuss. (**C10**, physician, male)

Some addressed the patient’s complaints in detail.

I am sorry that you are disappointed that I did not increase oxycodone as much as you wanted but it is a medication with serious side effects and can be dangerous. I have ordered appropriate imaging studies and as you have had these conditions for quite some time, these tests were not urgent and will be done fairly soon. Thank you for your service to our country. (**C9**, physician, male)

Others instead directed the message toward a potential future conversation. As one participant explained, “My whole purpose here is not to really fix all of the questions and answers, I'm just trying to diffuse his frustration. That’s the goal when I start typing it.” (**C6**, physician, female) Another explained his rationale for not answering every complaint, saying, “I’m not their therapist, I’m their doctor. And so I try to handle it from just a medical perspective.” (**C12**, physician, male)

Participants also displayed varying levels of empathy in their responses. Although the following message from a clinician indicated “I’m sorry,” the message was short.

I am sorry that you feel this way. Please make an appointment so we can best address your concerns. (**C10**, physician, male)

In contrast, the message below conveyed more empathy, and acknowledged hearing the patient. Additionally, this clinician was specific about how an appointment would address the issue, rather than just suggesting that an appointment be made.

I am sorry you are not happy with your care. There are a number of concerns expressed in your message so I think it would be best to have you schedule another appointment to see me to address each one. This may need to be done over several appointments in order to cover everything in a comprehensive manner. My nurse will call you to schedule an appointment. (**C13**, physician, female)

Participants expressed varied perspectives about secure messaging. The messages that they composed reflected such differences. Table 2 summarizes their perspectives on topics according to appropriateness for messaging. Participants found messaging useful, especially in shortening patient wait times for responses. At the same time, they felt that structured guidelines could help patients and clinicians alike. One commented, “My experience is that patients [try to be] very appropriate. [But they] don’t realize what is appropriate for messaging and what is not.” Despite this, few participants reported routinely discussing appropriateness with patients or otherwise educating them about messaging. Those who did described explaining that messaging is not meant for urgent situations. As for guidelines for clinicians, one articulated the need, saying, “I have my own system for when I would call or not call. There’re some obvious situations. But it would be helpful overall to have guidelines.” (**C16**, physician, female)

**Table 2.** Clinician attitudes on the appropriateness of using secure messaging to respond to patient messages of different topics

Topic or characteristic	Consensus: appropriate	Consensus: inappropriate	Disagreement
Routine lab results	x		
Routine medication questions	x		
Multiple issues in a message		x	
Urgent issues		x	
Emotionally charged			x
Complex			x
Sent from a non-designated family member			x

## DISCUSSION AND CONCLUSION

### Discussion

We identified situations that clinicians found were inappropriate for a secure messaging response (as in the case of a patient with chest pain and another new mention of depression that may require immediate action), and those were appropriate (such as a message about a routine laboratory result and one about routine medications). Clinicians differed in their assessments about the appropriateness of messaging for complex medication questions and an angry patient. Clinicians also felt that messaging should be used if there are only one or two issues to address. Longer, more complicated messages should be followed up by telephone, although the study did not explore thresholds of what was too long or complicated. Clinicians recognized that although urgent issues were inappropriate for secure messaging, patients may have difficulty assessing medical urgency. Likewise, they may also have difficulty in assessing what is emotionally appropriate to convey via secure messaging. Clinicians expressed varying levels of comfort for responding to emotionally charged concerns.

The content of clinicians’ secure messages in our study was neutral in tone and respectful in nature, consistent with prior evaluations.^2^^,^^12^ As well, some engaged in communication beyond information exchange, to respond to psychosocial information, and build relationships.^12^ Our findings were also consistent with those of Shimada *et al*,^14^ who found that clinician messages did not always resolve patients’ issues. In our study, whether participants addressed all issues posed in messages depended on both the clinician and the message content. One singular contribution of this study comes from conducting interviews with clinicians, and better understanding their thinking about their messages’ content and tone. The interviews elucidated the reasoning behind limiting responses to only “what is useful”, or responding to personal details to build relationships.

Patient–clinician communication is reciprocal, bi-directional, and context-dependent. The strength of the patient–clinician relationship can be influenced by participant traits, such as race, age, and gender; actions, such as active listening; and disclosing personal information.^15–18^ Because of these different factors, and the importance of context, the differences observed in clinician messages may differentially affect the patient–clinician relationship. While the reasons for differences in messages composed by clinicians may be stylistic, philosophical, or practical, they may have great consequences. Differences in how clinicians interpret patients’ concerns or perceive messages’ urgency and appropriateness may lead to different courses of action, which may lead to different outcomes. One clinician’s willingness to address something over messaging versus another’s reluctance may mean differences in speed of resolution, or the degree to which a patient felt heard, or whether a patient received accurate instructions. Patient preferences were beyond the scope of this study but should be investigated. Clinicians’ varying ways of interpreting and responding to a standardized set of messages in this study, as well as their patient anecdotes, suggest that patients, too, may have their own interpretations and preferences for the tone of received messages. Such preferences or personal styles of communication may be important for clinicians to consider when they compose messages.

Although this study focused on clinicians’ approaches to messages’ content, no participants indicated that messaging workload was unmanageable, regardless of message volume. While much has been written about the impact of the electronic documentation burden, less is known about messaging’s impact on clinician workload. Clinicians have sometimes considered messages’ added work to be a barrier, although most have indicated that secure messaging can improve care and safety.^19^ North *et al*^20^ found that messaging was not associated with in-person visit frequency.

### Implications

Clinicians and patients want and need greater guidance about secure messaging. In Table 3, we summarize the types of issues raised by participants but not yet addressed by existing guidelines. Patients are not being taught about messaging systematically, and discussions about messaging do not seem to occur routinely in primary care visits. Although hospitals may provide user guides for patients, the availability, amount, and quality of information vary across institutions.^21^ Future research and education efforts are needed to address the guideline needs and to examine the most effective methods for conveying how and when to use secure messaging most effectively.

**Table 3.** Issues to be addressed in guidelines about secure messaging

Topic	Sample considerations
Complexity	Number of issues Issues’ clinical nature
Urgency	Understanding of urgency for patients More immediate modes of communication available to patients
Relationship	Familiarity, trust, and other aspects of existing relationships that may affect communication Access to messaging by family members
Emotional content	Message’s purpose Recipient’s comfort in handling emotional content Safety

#### Limitations

The simulated nature of this study means that the messages that participants composed may not reflect how they actually communicate with patients. Vignettes simplify clinical cases and relationships, and may not fully encompass all factors that influence responses. Participants, however, reported that the study messages and vignettes were similar to ones they see in their practice, and the triangulation of participants’ messages with interview responses suggests that the messages reflect their approaches to messaging. In aggregate, this study design allowed comparisons across clinicians. The study sample was from two very specific healthcare systems. As such, results might not be applicable to clinicians in other healthcare systems, particularly those that approach secure messaging differently (eg, provide protected time to respond to these messages). However, we addressed this by recruiting primary care clinicians from two health systems with two different messaging systems. In seeking a range of perspectives, and gathering data until we reached theme saturation, we collect and present clinician experiences and rationale that may reflect a broad audience.

### Conclusion

This study highlights areas of disagreement in how clinicians respond to patients’ secure messages. Even as alternatives to in-person visits increase, clinicians and patients lack specific and circumstantial guidelines to navigate messaging. Therefore, institutions that govern and guide patient and clinician behavior, such as health systems, hospitals, and professional societies, should create guidelines and curricula to address these needs. This practical element of informatics education may differ for different circumstances. Understanding what is urgent, for example, may be easier than delineating the types of emotional content that are appropriate for messaging. More research is needed on effects on relationships, perceived quality of communication, satisfaction with care, medical errors, and other outcomes.

## FUNDING

This project was funded in part by the Indiana Clinical and Translational Sciences Institute, by grant numbers UL1TR001108 and UL1TR002529 from the National Institutes of Health, National Center for Advancing Translational Sciences, Clinical and Translational Sciences Award. The funders had no involvement in the design, analysis, and dissemination of the study.

## AUTHOR CONTRIBUTIONS 

MW is Chief of Health Services Research and Development at the Richard L. Roudebush Veterans Affairs Medical Center in Indianapolis, Indiana. The views expressed in this article are those of the authors and do not necessarily represent the views of the U.S. Department of Veterans Affairs. The Authors would like to express our gratitude to Rachel Gruber for her comments and copyediting that greatly improved the manuscript.

## SUPPLEMENTARY MATERIAL

Supplementary material is available at *Journal of the American Medical Informatics Association* online.

## CONFLICT OF INTEREST STATEMENT

The authors report no conflicts of interest.

## Supplementary Material

ooaa051_Supplementary_Data
