# Supplementary material for: Insecure messaging: how clinicians approach potentially problematic messages from patients
Source: JAMIA Open. 2020 Dec 5;3(4):576–82. doi: 10.1093/jamiaopen/ooaa051 (PMC7969962; doi:10.1093/jamiaopen/ooaa051)
Supplement: ooaa051_Supplementary_Data [file ooaa051_supplementary_data.docx]

**Appendix: Standardized vignettes and messages**

**Vignette 1. Anne Stafford**

Anne Stafford is a 56-year-old woman. You have been treating her for 3 years and share a friendly rapport. You saw her a few days ago for a routine appointment that included a discussion about her cough and about cholesterol. Her blood work shows that her cholesterol level is high: total cholesterol is 250, with LDL 150. She is not yet on a statin drug. Her ten-year cardiovascular risk is estimated to be 21%.

**Secure message 1**

From: Anne Stafford

Subject: Prescription

Doc,

How’d my blood work turn out? That cough medicine you gave me seemed to work. I don’t cough anymore but also think that we found the cause. I seemed to wheeze more when we had a fire in the fireplace. I have not used the inhaler for a few weeks because I have been visiting my daughter and her baby (my first grandkid!) and she doesn’t have a fireplace.

If you think I should have a prescription I’ll send you the number. Enjoyed our last appointment.

Thanks.

Anne

**Vignette 2. Evan Langley**

Evan Langley is 32-year-old man, and a relatively new patient in your practice. He started seeing you last year, when you treated him for bronchitis. The last time you saw him, about a month ago for a follow-up, he mentioned in passing that he was going through “a rough patch.” He has no documented history of depression.

**Secure message 2.**

From: Evan Langley

Subject: Next appointment

I need you to prescribe me something for depression Prozac something My financial situation Is Breaking Me Down Really bad

**Vignette 3. William Frank**

William Frank is a 73-year-old man; he has been your patient for 7 years. He is currently on diltiazem for hypertension; you recently decreased his dose because he was hypotensive and light-headed. You last saw him two weeks ago. His wife occasionally accompanies him to his appointments, although she wasn’t at the last one.

**Secure message 3.**

From: William Frank

Subject: Next appointment

Hi Doctor [ ], I am sending this message to ask you if there was a reason that you dropped William’s medicine from 240 to 180 and also took away his morphine. He is in serious pain and without the normal dose it makes it VERY hard to keep him comfortable and calm. He thought that when he talked to you recently that you were going to fix this incorrect amount and send him what he needed to take the normal dose of 1 every 3 hours. The last bottle dosage had him taking 2 less pills a day which was not enough to keep him comfortable. Please respond ASAP and let him know what you can do for him. I appreciate your care and concern, Thank you

Sincerely,

Linda Frank

**Vignette 4. Samuel Eisenberg**

Samuel Eisenberg is a 45-year-old man and has been your patient for 2 years. He has chronic back and neck pain, and difficulty walking.

**Secure message 4.**

From: Sam Eisenberg

Subject: Next appointment

Dr. [ ];

    I am rather concerned, that after providing you with my Medical Records, as well as, the detailed conversation about my medical symptoms, and my care at Eskenazi, that absolutely nothing has been done concerning what we discussed. And if your thoughts, that raising my Oxycodone, a measly 2.5 mg per dose, would some how appease me, or all I was seeking, you are sadly mistaken, and am overwhelmed with disappointment.

   I truly thought that you where sincere, and, concerned with the medical problems, and some severe symptoms, I am experiencing. You Distinctly told me that you would refer me for a Corroded Doppler test, and, a Heart and Lung Scan. As well, referral for an MRI of my Neck, Shoulder, and, upper to lower Back. C-1 through C-7 (Cervix) T-1 through T-12 ( Thoracic) L-1 through L-5 ( Lumbar) and S-1 through S-3 (Sacrum). Seeing that I have had a total of 5 surgeries, concerning the L4, L-5, S-1, would think it would be a first priority, for this to be done. Especially after my automobile accident effecting my Cervix, and Thoracic Spine.

   Or, have you neither taken the time , nor, had the concern you showed at my appointment with you?! Just a way to get rid of the "Problem Patient"?

Or, again does it come down to the money spent to do these things, and I receive free treatment, and no co-pay on anything?! Maybe you need a refresher course of your Hippocratic Oath! First of which is to do no HARM. Lack of treatment equals the same!

 Respectfully;

Sam Eisenberg

**Vignette 5. Howard Stanley**

Howard Stanley is a 64-year-old man; he has been your patient for 8 years. Although you only tend to see him for annual check-ups, you share a good rapport.

**Secure message 5**

From: Howard Stanley

Subject: Sudden chest pain

Dr. [ ],

I’m out west hiking near Seattle again. Earlier today, while on the hike, we were not up high, but I felt dizzy and had trouble catching my breath. My chest really hurt. My wife wanted me to stop. We finished dinner about an hour ago, and I feel OK at the moment. Should I be concerned? This has never happened before.

Best,

Howard

**Would you have called this patient by phone? (Yes or No)**

**Either way, please compose a secure message response below.**
